# Study protocol for a pilot randomized controlled trial to increase COVID-19 testing and vaccination among people who inject drugs in San Diego County

**DOI:** 10.1186/s13722-022-00328-z

**Published:** 2022-09-05

**Authors:** Angela R. Bazzi, Alicia Harvey-Vera, Tara Buesig-Stamos, Daniela Abramovitz, Carlos F. Vera, Irina Artamonova, Thomas L. Patterson, Steffanie A. Strathdee

**Affiliations:** 1 Herbert Wertheim School of Public Health, University of California, 9500 Gilman Drive, La Jolla, San Diego, CA MC0631 USA; 2 Department of Community Health Sciences, School of Public Health, Boston University, 715 Albany St, Boston, MA USA; 3 School of Medicine, University of California, 9500 Gilman Drive, La Jolla, San Diego, CA MC0507 USA; 4 Facultad de Medicina, Universidad Xochicalco, Rampa Yumalinda 4850, Chapultepec Alamar, 22110 Tijuana, B.C Mexico; 5 United States-Mexico Border Health Commission, Paseo del Centenario 10851, Zona Urbana Rio Tijuana, 22320 Tijuana, B.C Mexico; 6 OnPoint, Harm Reduction Coalition of San Diego, 1389 Windmill Road, El Cajon, CA USA; 7 Department of Psychiatry, University of California, 9500 Gilman Drive, La Jolla, San Diego, CA MC0680 USA

**Keywords:** Substance use, Intravenous, SARS-CoV-2, COVID-19 testing, Vaccination, Vulnerable populations, Harm reduction, Motivational interviewing

## Abstract

**Background:**

People who inject drugs (PWID) have low rates of COVID-19 testing and vaccination and are vulnerable to severe disease. We partnered with a local, community-based syringe service program (SSP) in San Diego County, CA, to develop the single-session theory- and evidence-informed “LinkUP” intervention to increase COVID-19 testing and vaccination. This paper details the protocol for a pilot randomized controlled trial (RCT) of the LinkUP intervention.

**Methods:**

With significant community input into study design considerations, including through our Community and Scientific Advisory Board, the LinkUP pilot RCT leverages an ongoing cohort study with adult (≥ 18 years) PWID in San Diego County to recruit participants who have not recently undergone voluntary COVID-19 testing and are unvaccinated. Eligible participants are referred to SSP locations randomized to offer the active intervention (involving tailored education, motivational interviewing, and problem-solving strategies) or a didactic attention-control condition (information sharing only). Both conditions are delivered by trained peer counselors hired by the SSP and were designed to be delivered at mobile (outdoor) SSP sites in ~ 30 min. Intake data assesses COVID-19 testing and vaccination history, health status, and harm reduction needs (to facilitate SSP referrals). At the end of either intervention condition, peer counselors offer onsite rapid COVID-19 antigen testing and COVID-19 vaccination referrals. Out-take and follow-up data (via SSP and state health department record linkages) confirms whether participants received the intervention, COVID-19 testing (and results) onsite or within six months, and vaccination referrals (and uptake) within six months. Planned analyses, which are not powered to assess efficacy, will provide adequate precision for effect size estimates for primary (COVID-19 testing) and secondary (vaccination) intervention outcomes. Findings will be disseminated widely including to local health authorities, collaborating agencies, and community members.

**Discussion:**

Lessons from this community-based pilot study include the importance of gathering community input into study design, cultivating research-community partnerships based on mutual respect and trust, and maintaining frequent communication regarding unexpected events (e.g., police sweeps, neighborhood opposition). Findings may support the adoption of COVID-19 testing and vaccination initiatives implemented through SSPs and other community-based organizations serving vulnerable populations of people impacted by substance use and addiction.

*Trial registration* This trial was registered prospectively at ClinicalTrials.gov (identifier NCT05181657).

## Background

COVID-19 testing and vaccination are essential components of the national pandemic response, particularly with the emergence of new SARS-CoV-2 variants. However, pronounced disparities in testing and vaccination uptake persist in many U.S. communities. People with substance use disorders, including people who inject drugs (PWID), are vulnerable to SARS-CoV-2 infection and severe disease yet have suboptimal rates of COVID-19 testing and vaccination [1–8]. Studies by our team and others have identified multilevel barriers to COVID-19 testing and vaccination among PWID, including low COVID-19 knowledge and perceived risk, institutional mistrust, addiction-related stigma, and other structural barriers to healthcare access and utilization (e.g., homelessness, limited transportation) [9–13]. Preliminary analyses from our ongoing, binational cohort study in the San Diego-Tijuana border region found that one third of PWID in San Diego County had been infected with SARS-CoV-2 as of June, 2021; among these individuals, two thirds had never been tested for COVID-19, and only 3% had been fully vaccinated against COVID-19 [8].

PWID often prefer receiving health and prevention services and referrals in community-based venues outside of the formal healthcare system, including through syringe service programs (SSPs) whose staff are viewed as trusted sources of information and support [14–18]. Many U.S. SSPs have the capacity to deliver essential preventative services such as HIV and Hepatitis C virus (HCV) testing, and HBV vaccination [19–24]. Some SSPs can also engage and retain PWID in healthcare services provided onsite, including HCV and opioid use disorder (OUD) treatment [25–27], supporting the role of SSPs as critical “touchpoints” for reaching marginalized PWID who are not engaged in the formal healthcare system. While mobile SSPs likely reach even further into vulnerable communities of PWID than “brick and mortar” SSPs [28], they often have limited capacity to directly provide healthcare services to PWID. Nevertheless, many mobile SSPs provide supported referrals to healthcare and OUD treatment services and can increasingly connect their participants to these services via telemedicine [29]. In the COVID-19-pandemic era, a survey exploring U.S. SSPs’ capacity to directly provide vaccination services identified important barriers included staffing needs (e.g., personnel licensed to administer vaccines), supply and storage challenges, safety concerns, competing priorities, and limited systems to support follow-up for multidose vaccines [30]. For these reasons, despite the ability of many SSPs to offer some essential preventative services onsite [31], more research is needed to optimize their potential role in COVID-19 testing and vaccination [32].

In response to the growing body of evidence on barriers to COVID-19 testing and vaccination among PWID, and the success of SSPs as a prevention touchpoint engaging this population, we developed the SSP-based “LinkUP” intervention to increase COVID-19 testing and vaccination in an underserved population of PWID in San Diego County, CA. LinkUP is informed by Social Cognitive Theory (SCT), which has shown promise in supporting HIV treatment and prevention interventions in socially-marginalized substance using populations [33–35] and posits that knowledge, motivation, self-efficacy, and behavioral skills can support individuals in overcoming social and structural barriers [36]. As such, LinkUP uses education, motivational interviewing (MI), and problem-solving and planning strategies to increase COVID-19 testing and vaccination knowledge, motivation, self-efficacy, and behavioral skills [37, 38]. It is delivered by MI-trained peer counselors hired by a mobile SSP providing onsite rapid COVID-19 testing and vaccination referrals to nearby sites. Herein, we describe the procedures for a randomized controlled trial (RCT) piloting the LinkUP intervention (ClinicalTrials.gov identifier NCT05181657).

## Methods

### Objectives and hypotheses

The overall objective of the LinkUP pilot RCT is to determine preliminary efficacy and effect sizes of the active intervention (compared to an attention-control didactic condition) in increasing the uptake of COVID-19 testing (primary outcome). A secondary objective is to assess whether LinkUP can increase the uptake of COVID-19 vaccination (secondary outcome). Based on SCT and preliminary evidence, we hypothesize that LinkUP will increase COVID-19 testing and vaccination uptake by increasing COVID-19 testing knowledge and motivation while reducing structural barriers to access.

### Overview of trial design and study setting

LinkUP represents a partnership between investigators at the University of California, San Diego (UCSD), and the OnPoint SSP of the Harm Reduction Coalition of San Diego. Established in 2018, OnPoint is the only mobile SSP in San Diego County, and the only program currently operating on a needs-based distribution policy, meaning that, in line with California Department of Public Health (CDPH) guidelines, it provides syringes and other harm reduction supplies to its participants based on their needs rather than restrictive access policies (e.g., “one for one exchange” policies) that limit the provision of syringes per transaction [39]. OnPoint distributes harm reduction services, including safer injecting, smoking, and sex supplies, naloxone and fentanyl test strips, and supported referrals to health and social service agencies, to people who use drugs (including PWID) across San Diego County. These services are provided by OnPoint’s harm reduction specialists, who are cross-trained in MI and case management, and a network of volunteers, many of whom have lived experience with substance use and homelessness.

LinkUP is nested within the ongoing, binational “La Frontera” cohort study of PWID in the San Diego-Tijuana border region (R01 DA049644; PI: Strathdee). This parent study was funded on April 1, 2020, and aims to study incidence and predictors of HIV, HCV, and overdose in relation to cross-border mobility and drug market trends through 2025. To be eligible for La Frontera, individuals aged ≥ 18 or older who injected drugs within the last month and reported living in San Diego County (n = 400) or Tijuana, Mexico (n = 200) were recruited through street outreach, as previously described [8]. Among San Diego County participants, 200 were required to have crossed the border to inject drugs in Mexico within the previous two years. At baseline and semi-annually, participants undergo interviewer-administered surveys and provide blood specimens for HIV and HCV testing. Recruitment for La Frontera began in October 2020.

In May 2020, La Frontera investigators received additional funding to study the prevalence and correlates of SARS-CoV2 infection in the cohort. Preliminary data showing high SARS-CoV2 seroprevalence and low COVID-19 testing and vaccination uptake informed the design of the LinkUP pilot intervention [8, 11, 13], which was funded in September, 2021, through the RADx® Underserved Populations (RADx-UP) initiative of the National Institutes of Health (NIH), which was created “to ensure that all Americans have access to COVID-19 testing, with a focus on communities most affected by the pandemic” [40]. Since RADxUP funding was restricted to U.S. projects, LinkUP had the goal of enrolling 150 participants into a pilot RCT focused only on La Frontera cohort participants living in San Diego County. All study procedures for the La Frontera parent study and LinkUp intervention pilot study were reviewed and approved by the institutional review boards (IRBs) of the University of California, San Diego. All participants in La Frontera and LinkUP provided written informed consent.

### Eligibility criteria

Individuals may be eligible for the LinkUP intervention pilot study in two ways. First, eligibility includes [1] being enrolled in La Frontera (and residing in San Diego County); (2) reporting not having ever been voluntarily tested for COVID-19 outside of La Frontera or reporting having had a mandatory COVID-19 testing (e.g., as required for incarcerated persons by the California Department of Corrections and Rehabilitation; CDCR) [41] over two months ago; and (3) meeting the study’s “unvaccinated” definition (see Table 1). Alternatively, eligibility includes [1] being enrolled in La Frontera (and residing in San Diego County); and (2) meeting the study’s “symptomatic” definition which was based on criteria from the U.S. Centers for Disease Control and Prevention [42] (Table 1). For the latter, we placed less emphasis on symptoms that could be due to opioid withdrawal (e.g., body aches, fatigue, headache) and greater emphasis on symptoms consistent with acute SARS-CoV-2 infection (i.e., fever, chills, sore throat). Participants must also provide consent to release their medical records to the study team, and consent to share their de-identified data with the RADxUP Data Coordinating Center at Duke University.

**Table 1:** Study definitions for “unvaccinated”, partially vaccinated and “symptomatic” inclusion criteria

Inclusion Criterion	Definition
Unvaccinated	• Never vaccinated against COVID-19
Partially Vaccinated*	• Received only 1 dose of the Moderna^®^ COVID-19 vaccine ≥ 1 month ago or only 1 dose of the Pfizer^®^ COVID-19 vaccine ≥ 3 weeks ago; OR • Received only 1 dose of the Jenssen^®^ COVID-19 vaccine ≥ 2 months ago; OR • Received 2 doses of the Moderna^®^ or Pfizer^®^ COVID-19 vaccines with the last dose received ≥ 5 months ago; OR • Received 1 dose of another vaccine ≥ 1 month ago; OR • Received 2 doses of another vaccine with the last dose received ≥ 5 months ago
Symptomatic	• Reporting having fever/chills within the last week without having tested positive for COVID-19 within the last month; OR • Reporting having shortness of breath within the last week without having tested positive for COVID-19 within the last month; OR • Reporting ≥ 2 of the following COVID-19 symptoms: a. New loss of smell or taste b. Cough c. Sore throat, congestion, or runny nose d. Skin rash within the last week without having tested positive for COVID-19 within the last month

* Participants reporting being vaccinated but not remembering which vaccine they received are classified as partially vaccinated (e) if they only received 1 dose or (f) if they received 2 doses

### Screening and enrolment

We initially obtained information on potential eligibility by reviewing La Frontera survey data. Then, for those who indicated interest in being re-contacted for future research studies, we contacted participants through phone calls, texts, Facebook Messenger and street outreach to assess their LinkUP eligibility using a short screener to confirm that participants’ COVID-19 testing and vaccination status had not changed. Of the 400 San Diego La Frontera participants who had been recruited by September 2021, approximately 100 were deemed potentially eligible for LinkUP by the time the pilot RCT was ready to begin. Therefore, we sought approval from UCSD’s IRB and NIH to re-open recruitment for La Frontera to ensure that the target sample size for LinkUP could be met. These additional participants were enrolled through street outreach. Where possible, efforts were made to recruit participants in the same locations and the same times when SSP staff were conducting mobile syringe exchange and naloxone provision. Trained, bilingual La Frontera staff obtain written informed consent using forms available in English and Spanish. The consent form includes optional permission to share de-identified data between the OnPoint SSP and the UCSD research team to permit future record linkage to confirm future COVID-19 vaccination status. LinkUP began enrollment in March 2022.

### Baseline assessments

Being nested within La Frontera, LinkUP can leverage the parent study’s extensive cohort assessments for socio-demographic and behavioral data. La Frontera baseline and follow-up electronic surveys in English and Spanish were programmed using Questionnaire Development System (QDS) and installed on laptop computers. Trained, bilingual interviewers administer these surveys to the study participants using computer-assisted interviewing. Since recruitment for La Frontera has taken place during the COVID-19 epidemic, most baseline and follow-up interviews to date have been conducted outdoors under canopies near the study van with physical distancing and facemasks for interviewers and participants, as specified in the parent study protocol [8]. All data collection has been performed in the community, at various locations around San Diego County, including in homeless encampments, canyons, vacant lots, and other public areas (e.g., parks, areas adjacent to homeless shelters).

La Frontera baseline survey domains include socio-demographics, mobility patterns, injection and non-injection drug use behaviors, sexual behaviors, incarceration, homelessness, and healthcare utilization. Follow-up surveys cover similar domains with a 6-month recall period for most measures. To accommodate the addition of COVID-19 related measures and to reduce participant burden, we administer a supplemental COVID-19 survey to all participants one week after the baseline assessment. This supplemental survey assesses COVID-19 exposures, protective behaviors, social networks, misinformation and disinformation, and additional measures from the RADx-UP Tier 1 Common Data Elements [43]. LinkUP participants with their most recent La Frontera interviews over three months ago undergo brief interviewer-administered surveys to reassess potential barriers to COVID-19 testing and vaccination and update the supplemental survey data described above.

Blood samples are drawn at all La Frontera study visits for HIV and HCV testing [8]. Participants with COVID-19 symptoms are referred to local community health clinics. Participants receive $20 to reimburse their time and the cost of transportation and a laminated photo ID card embossed with the La Frontera logo to which stickers with the LinkUP logo are added. Participants are then referred to nearby OnPoint SSP sites that have been allocated to provide either the active or didactic LinkUP condition on a given week, as described below, for which they received an additional $10 compensation regardless of whether or not they choose to undergo COVID-19 testing or vaccination.

### Randomization and intake

We initially considered randomizing SSP locations to deliver either the active intervention or didactic control condition (described below) to reduce the potential for contamination and help ensure that no neighborhood in San Diego County was denied access to the intervention. However, due to mobility of the study population and difficulty determining in advance how many locations would be suitable when the trial began, creating a sampling frame by SSP location was ultimately not possible. Instead, we randomized the weeks of study implementation to involve either intervention or didactic condition administration (i.e., during a particular week, OnPoint and the study team administer which condition was allocated to that week, regardless of SSP location).

Efforts are made to co-locate staffing and field operations so that participants consented for LinkUP can be immediately seen by OnPoint peer counselors. OnPoint peer counselors confirm that individuals are LinkUP participants by viewing the laminated ID card provided by study staff. Peer counselors then conduct a brief, five-minute interview to re-assess COVID-19 testing and vaccination history, health status, and harm reduction needs (to facilitate referrals to other services). Following intake interviews, participants either receive the LinkUP active intervention or didactic control condition according to the randomized allocation of the SSP site that week.

### Active intervention condition

The active condition involves a single-session, manualized intervention that we developed based on literature review, formative research [8, 12, 13], and consultations with our Community and Scientific Advisory Board (CSAB) and collaborating SSP research partners. Aligned with SCT [36], key intervention strategies involve tailored education, MI, and problem-solving and planning around individual participants’ unique concerns about and barriers to COVID-19 testing and vaccination. Following review and approval by our CSAB and Data Safety and Monitoring Board (DSMB), bilingual, bicultural study staff translated and back-translated all intervention materials into Spanish. The active intervention session was designed to last approximately 30 min on average.

Although the intervention session was designed to be interactive and flexible, specific intervention components and strategies are detailed in the manual and include the following, as shown in Table 2. First, peer counselors provide basic COVID-19 testing and vaccination education using several brief educational videos, available in English and Spanish, that include facts on COVID-19 transmission, testing, and vaccination (totaling approximately 5 min). To help tailor the educational content to participants’ needs, counselors are trained to answer questions on COVID-19 epidemiology, testing, vaccines (including booster shots), and available treatments using information drawn from CDC guidance and literature shared by our RADxUP consortium leaders. Rather than lecturing, peer counselors then use key MI techniques [37, 38] to engage participants in discussion about evidence-based COVID-19 information, misinformation (e.g., “COVID-19 is no worse than the flu”), and disinformation identified in our preliminary work (e.g., “COVID-19 vaccines include a government chip or tracking device” or “can alter one’s DNA”) [9–13]. Next, peer counselors attempt to identify participants’ primary concerns about COVID-19 testing and vaccination to tip their decisional balance. Counselors then engage participants in problem-solving around specific barriers to future testing and vaccination. Finally, at the end of the session, counselors offer participants onsite COVID-19 testing and referrals for vaccination (described below).

**Table 2:** LinkUP intervention targets, strategies, and specific activities

Intervention targets	Intervention strategies	Examples of specific activities	Format
Limited COVID-19 knowledge	Basic COVID-19 education	• General background on COVID-19 including symptoms • Facts on COVID-19 testing • Facts on COVID-19 vaccination (primary series and booster shots) • COVID-19 treatment information • Provision of local resources for testing and vaccination	Brief educational videos on COVID-19, testing, and vaccination (and CPR for attention-control didactic condition); appendix of local resources
Limited COVID-19 testing and vaccine motivation	Motivational interviewing	• Assessing readiness for COVID-19 testing and vaccination • Decisional balance scales (i.e., rating personal motivation) for COVID-19 testing and vaccine • Assessing individuals’ specific COVID-19 concerns • Debating and clarifying COVID-19 (mis)information (e.g., vaccine myths vs. facts) • Reviewing local COVID-19 situation and adding personal meaning	Personalized discussion informed by the principles of motivational interviewing (with reflection, affirmation, normalization)
Limited COVID-19 testing and vaccine self-efficacy and behavioral skills	Problem-solving and planning	• Identifying personally-relevant challenges to COVID-19 testing and vaccination (e.g., competing priorities, scheduling appointments when required, homelessness, transportation, etc.) • Planning for future COVID-19 testing and vaccination (listing anticipated plans, challenges to plans, and solutions)	Personalized discussion with problem-solving and planning around specific structural and social barriers
COVID-19 testing and vaccine update	(d) Offer services and referrals	• Offer on-site rapid COVID-19 antigen testing or PCR sample collection • Offer referrals for COVID-19 vaccination at nearby clinics and pharmacies	Personalized discussion

### Didactic attention-control condition

The didactic condition, also manualized, is delivered by peer counselors and includes the same brief educational videos (in English and Spanish) on COVID-19 transmission, testing, and vaccination that are utilized in the active intervention condition (totaling approximately 5 min). As the didactic session was intentionally designed to last approximately 30 min as an attention-control condition, counselors then show participants an educational video on material relevant for PWID (e.g., cardiopulmonary resuscitation; CPR) lasting approximately 25 min. Peer counselors are instructed to offer participants on-site rapid COVID-19 testing (described below) and provide information on COVID-19 testing, vaccines including booster shots, available treatments, and referrals to nearby testing and vaccination services, if desired, using standard scripts and a resource appendix. Although counselors do not engage in MI counseling at any point within the didactic session, they can answer questions that participants have and, at the end of the session, offer participants onsite COVID-19 testing and vaccination referrals (described below).

### Interventionist training and supervision

Peer counselor training includes formal training in human subjects’ research ethics, up to eight hours of self-directed MI training (depending on their level of experience), and a three-hour seminar on COVID-19 biology, testing, vaccination, and common misinformation and disinformation. Peer counselors are also given structured opportunities to observe and practice using the intervention manuals (in pairs, with feedback from the research Project Director) and administering rapid COVID-19 tests. The PhD-level Project Director, who is experienced in MI and regularly reviews scientific updates on COVID-19 biology and public health recommendations regarding COVID-19 testing and vaccination, supervises intervention delivery on a daily basis. She meets one-on-one with peer counselors to provide refresher training and feedback to help ensure fidelity to the intervention manuals and to update counselors on new vaccines that become availableWe are also monitoring peer counselors’ fidelity to the intervention manuals using structured checklists.

### Onsite COVID-19 testing

Immediately following completion of the didactic or active intervention conditions, peer counselors offer participants onsite rapid COVID-19 antigen testing (BiNaxNow^®^) [44]. For participants who agree to testing, peer counselors provide brief pre- and post-test counseling and instruct participants on how to self-collect nasal swabs following manufacturer instructions; results are read and shared with participants within 15 min. Those testing positive are asked to provide an additional nasal swab for PCR confirmation at a CDPH-certified lab. Participants are also permitted to have a PCR test instead of a rapid test, if desired, in which case they are asked to provide contact details to permit counselors to share their test results with them later. Participants testing positive on either or both rapid and/or PCR tests are advised to practice physical distancing and wear facemasks. Furthermore, any participants testing positive or exhibiting symptoms consistent with COVID-19 are referred to nearby community clinics for free medical care. Neighborhood-specific lists of other community resources have been compiled to facilitate referrals to other community services as appropriate.

### COVID-19 vaccination referrals

Upon completion of the active intervention or didactic conditions, peer counselors also refer interested participants for COVID-19 vaccination at nearby clinics and pharmacies offering FDA-approved or -authorized vaccines. Participants who express interest in vaccination are given a piece of paper with the names, locations and phone number of community vaccine clinics as well as nearby pharmacies that are offering vaccinations. The list of referrals was created by verifying each site's location, phone number, hours of operation and requirements before being included in the list of referrals. The list not only included San Diego Public Health locations, community clinics but also pharmacies that were offering COVID vaccinations with or without appointments. We also indicate which pharmacies agreed to vaccinate on a walk-in basis without an appointment. Additionally, regular communication with our CSAB has supported our study team in identifying local public health initiatives involving COVID-19 vaccination that would be available to participants (e.g., “pop-up” vaccine drives run by CDPH and “health fairs” implemented by community health centers).

### Post-intervention assessments and outcome ascertainment

After study visits, peer counselors supplement the intake data by completing an out-take data collection form assessing whether or not participants received the LinkUP intervention, the time taken, whether the participant agreed to receive a COVID-19 test(s), and their rapid test results and vaccination referrals. These data are shared through record linkage between OnPoint and the La Frontera parent study team to confirm study outcomes. Record linkage will also be conducted with CDPH’s COVID-19 database based on electronic health records of participants providing a release of medical information to confirm the results of COVID-19 PCR tests (if and where subsequent COVID-19 testing was done elsewhere over six months of follow-up) and whether participants received COVID-19 vaccines, including the vaccination types, dates, and numbers of doses (over six months of follow-up). Finally, record linkage will also be used to record any subsequent COVID-19-related hospitalizations or deaths.

### Sample size and planned data analyses

We anticipate enrolling 150 participants into the LinkUP pilot trial, which will provide adequate precision for effect size estimates but is not powered to assess efficacy. We will conduct an intent-to-treat (i.e., per-randomization) analysis following CONSORT guidelines [45]. We will compare socio-demographics across the two study arms and if any significant differences are found we will be controlling for potential confounders and study locations in all subsequent analyses. To obtain effect sizes and standard errors, percentages along with 95% CIs for those who (a) underwent COVID-19 testing onsite or within 6 months of the intervention and (b) had ≥ 1 COVID-19 vaccine dose within 6 months of the intervention, respectively, will be calculated for the two study arms. We decided upon a 6 months’ time frame for assessing outcomes after discussing this issue with our CSAB members, who felt that some participants might delay their decision to be vaccinated, but still be influenced by the intervention for this period of time. In previous intervention studies, intervention effects waned after 6 months [46, 47].

To obtain risk ratio estimates (intervention/control), we will use logistic regression mixed models with outcomes for (a) having had a COVID-19 test onsite or within 6 months of the intervention (primary outcome), and (b) having had ≥ 1 COVID-19 vaccine dose within 6 months of the intervention. These models will treat intervention condition (active intervention vs. didactic control) as the primary fixed effect, and include potential covariates (e.g., age, gender, homelessness, income) and a random intercept for subject. In the analysis to study the effect of the intervention on COVID vaccination uptake (secondary outcome), we plan to control for “unvaccinated vs. partially vaccinated” as a baseline covariate.

### Timeline and dissemination plans

**Fig. 1:**
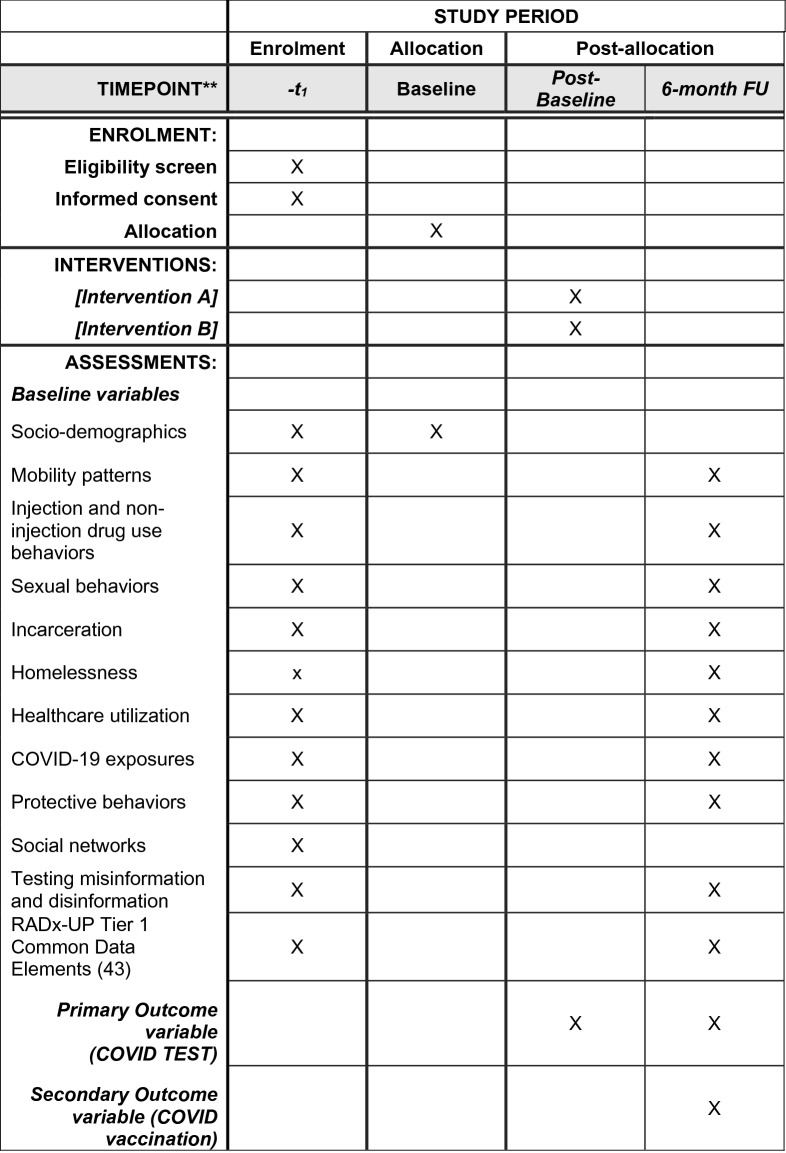
Schedule of enrolment, interventions, and assessments for LinkUP trial

Results from LinkUP will be shared with OnPoint and La Frontera staff, the CSAB, RADxUP consortium, state (CDPH) and local (San Diego County) health department officials and other policymakers and program planners to help inform efforts to reduce health disparities related to COVID-19. With support from OnPoint personnel and CSAB input, we will also determine the best method for sharing study results back with the local PWID communities in each of the primary study and SSP sites. Effect sizes estimates may also inform subsequent intervention research, including a fully powered efficacy trial, with this population and setting (see Fig. 1).

## Discussion

Our team of researchers and service providers learned several important lessons from the design and execution of this community-based RCT to improve COVID-19 testing and vaccination uptake among PWID in San Diego County. First, in the context of a dynamic and ongoing pandemic, it is critical to keep abreast of changes in pathogen epidemiology that can influence recommendations from health and regulatory agencies such as the CDC and Food and Drug Administration (FDA). When our protocol was originally submitted for funding in May 2021, SARS-CoV-2 rapid testing kits and vaccines were not yet available in community settings such as SSPs, so we developed contingency plans to account for different ways these interventions could be implemented by state and county health departments. By the time we received funding in September 2021, rapid antigen tests had become available to SSPs across California with state support, but logistical challenges prevented our community partner, the OnPoint SSP, from providing on-site COVID-19 vaccination. Our contingency planning helped our team pivot by identifying local community resources and facilitating referrals to vaccine clinics and pharmacies near the mobile SSP outreach locations. As most vaccination sites required individuals to present a government-issued ID card, a resource many of our participants lacked, we communicated with vaccine clinics and pharmacies in advance to ensure that they understood this barrier to healthcare access for vulnerable populations [48] and would agree to honor our La Frontera study-issued photo ID cards.

After our protocol received approvals from our IRB, DSMB, and the RADxUP consortium, we expected to begin the trial in early 2022 but were thwarted by unexpected challenges. New evidence on waning vaccine efficacy amidst the emergence of new SARS-CoV-2 variants prompted the CDC to update COVID-19 vaccination guidelines and recommend a booster shots [49]. In the meantime, we had completed formative research on barriers to COVID-19 testing and vaccination among PWID that led us to contemplate changes to our eligibility criteria. Specifically, we found that PWID who had been recently incarcerated or were homeless were significantly more likely to have undergone COVID-19 testing [13]. Consultations with our CSAB and in-depth interviews with PWID, supported with funding from the San Diego Center for AIDS Research, revealed that individuals in contact with the criminal justice system and homeless shelters were often subjected to mandatory (as opposed to voluntary) COVID-19 testing that had made them reluctant to engage in future voluntary testing [12]. Similarly, our original eligibility criteria would have excluded individuals with only one COVID-19 vaccine dose, and we had not considered that individuals with symptoms consistent with SARS-CoV-2 should be included in the trial irrespective of whether they had been previously tested or vaccinated. After consulting with our DSMB, CSAB, NIDA program officer, and RADxUP leadership about these issues, we decided that individuals who had received mandatory but not voluntary COVID-19 testing would be eligible provided that it was conducted at least two months ago. We also revised eligibility criteria to include those who were not fully vaccinated or reported past-week COVID-19 symptoms. Fortunately, these changes, which were well-aligned with current public health guidelines, were rapidly reviewed and approved, resulting in delayed initiation of the trial by only a few weeks.

A second important lesson was that partnerships between researchers and community-based service organizations like SSPs need to be cultivated based on shared responsibilities, mutual respect, and trust. Since members of our research team had worked with harm reduction service providers for many years, we were aware that our priorities are different. OnPoint staff appreciated that LinkUP data collection could ultimately benefit their participants. However, the syringes, naloxone, and other prevention supplies OnPoint distributed had a more immediate impact on their participants’ health compared to the potential benefits of our research, which were perceived to be much more distal and indirect. “You don’t worry about COVID-19 when you’re dead from an overdose,” one OnPoint staff member commented, continuing, “The needs of our clients come first. The research should wrap around the services, not the other way around.” As a result, we coordinated schedules between researchers and SSP staff to optimize both parties’ priorities. For example, to locate individuals eligible for the study, mobile units needed to gain access to areas that were unknown to most service providers (e.g., canyons, homeless encampments along highways, abandoned buildings). Before attempting to recruit participants in locations that were new for the SSP, we enabled OnPoint staff to conduct outreach at least a few weeks in advance, allowing them to provide critical prevention services, gain trust of the local community, and assess the potential of new sites for research activities.

Since our trial was designed to embed the MI intervention into OnPoint’s routine to enhance sustainability, peer counselors were hired and supervised by the SSP rather than the research team. With childcare and school schedules of these part-time staff, we ultimately hired and trained a larger team of peer counselors than originally planned so at least two counselors would be available each shift. Staff with lived experience needed additional schedule flexibility and support, both physical and emotional. When one counselor’s vehicle broke down, our research team supported her with transportation until her car could be repaired. Some counselors were nervous about their job performance. We learned that they gained confidence by practicing role plays, observing counseling sessions conducted by more experienced staff, and conducting the didactic control session first before attempting the active intervention session which required application of their MI skills. Patience was required on behalf of research and SSP staff; by clarifying expectations and creating open lines of communication (e.g., via the group chat function on a social media app), logistical problems could be overcome.

A third lesson was that unexpected events accompanied our mobile field operations, including heat exposure, police activity, and concerns from local community members. We had originally intended to recruit participants in a storefront office that would be supplemented by mobile outreach as needed. However, most of the locations we accessed were geographically dispersed, and there were ongoing concerns about SARS-CoV-2 transmission in indoor settings. We ultimately conducted almost all project activities (including recruitment, informed consent, interviewing, specimen collection, counseling, and rapid testing) outdoors. This required new standard operating procedures for what evolved into “pop-up” research sites, and re-budgeting to account for unanticipated items such as canopies, sandbags, extension cords and folding tables and chairs. Weekly staff meetings and the group chat helped troubleshoot these issues as they arose. For example, when the open nature of our data collection led some OnPoint clients to interrupt confidential study procedures, one staff member was appointed as a “lookout” to prevent such interruptions and protect participant confidentiality. We also purchased a back-up generator for the study van during a period of extreme heat to maintain an internet connection and refrigeration for study specimens.

Although inclement weather could often be anticipated, so-called “police sweeps” of homeless encampments were frequent but less predictable for our team. On more than one occasion, staff arrived for their shift at a prespecified location that had been a source of many eligible participants the day before, only to find that local police or transportation authorities had suddenly cleared all homeless encampments in the vicinity, disrupting study recruitment and the provision of critical prevention supplies such as naloxone, syringes, and other harm reduction equipment. Increasingly common across the United States, these sweeps or “clean-up” operations involve forcibly displacing persons experiencing homelessness [50, 51], including PWID, leading to numerous adverse outcomes including the loss of medications, prevention supplies, ID cards, and other personal belongings [52, 53]. To make matters worse, on more than one occasion, research staff were harassed by police. These circumstances required having back-up plans and establishing open lines of communication between police departments and investigators, leveraging relationships that SSP leadership had already cultivated with local authorities.

Another issue we encountered involved concerns from local community members with clear “not in my backyard” (i.e., “NIMBY”) sentiments. Following a week of data collection in a homeless encampment on the outskirts of a rapidly gentrifying neighborhood, the executive director of OnPoint received an email from a community member demanding to know what we were doing and expressing concerns that our activities were “attracting additional homeless to the neighborhood,” which the complainant felt would increase neighborhood crime. The OnPoint director and lead research investigator carefully drafted a response explaining the purpose of the harm reduction services and research activities along with related benefits for the local community. To allay additional concerns, our study logo was used to brand project vehicles and tents.

Overall, we believe that our design and operational changes in response to the issues described above, along with additional, smaller considerations, are contributing to the success of the LinkUP study. For example, one staff member brought a blanket and water bowl so peer counselors’ and participants’ pet dogs could wait comfortably during study visits. We had pizza delivered to the team on a particularly hot day when staff worked over-time to complete counseling for a final participant. Combined with frequent communication, these efforts generated a sense of teamwork and boosted morale, and over time, comments in the group chat transitioned from negative to positive, supportive notes of encouragement.

## Conclusion

In summary, our team’s experience implementing a community-based pilot study to improve COVID-19 testing and vaccination with marginalized PWID illustrates the importance of flexibility and teamwork to address unanticipated challenges. Public health initiatives for socially and structurally marginalized populations such as PWID may benefit from genuine collaboration with community-based organizations like SSPs, provided the research can wrap around the services and relationships are built on mutual respect and trust. Flexibility and responsiveness of funders and advisory bodies can also support the success of such initiatives, whether in pandemic times or beyond. Ultimately, it is our hope that findings and lessons from this pilot study will support the adoption of the LinkUP intervention and possibly other public health initiatives through SSPs and other community-based organizations serving vulnerable populations of people impacted by substance use and addiction.

## Data Availability

The protocol and statistical analysis plan are available from the corresponding author upon written request. The final dataset will be analyzed by investigators at the University of California, San Diego, and can be made available as de-identified data to investigators upon reasonable request with the required IRB approval and data use agreements.

## Abbreviations

CDC: Centers for Disease Control and Prevention
CDCR: California Department of Corrections and Rehabilitation
CDPH: California Department of Public Health
CPR: Cardiopulmonary resuscitation
CSAB: Community and Scientific Advisory Board
DSMB: Data Safety and Monitoring Board
FDA: Food and Drug Administration
HCV: Hepatitis C virus
IRB: Institutional review board
NIMBY: “Not in my backyard”
MI: Motivational interviewing
PWID: People who inject drugs
RCT: Randomized controlled trial
SSP: Syringe service program
SCT: Social Cognitive Theory
UCSD: University of California, San Diego
